# Low-dose radiation and malathion co-exposure instigates long-term neurological sequelae and synergistic disruption of lipid homeostasis and energy metabolism in the hippocampus

**DOI:** 10.1038/s41598-025-16175-2

**Published:** 2025-09-26

**Authors:** Rekha Koravadi Narasimhamurthy, Babu Santhi Venkidesh, Herman Sunil Dsouza, Manjunath B. Joshi, Thokur Sreepathy Murali, Shama Prasad Kabekkodu, Bola Sadashiva Satish Rao, Kamalesh Dattaram Mumbrekar

**Affiliations:** 1 Department of Radiation Biology & Toxicology, Manipal School of Life Sciences, Manipal Academy of Higher Education, Manipal, 576104 Karnataka India; 2 Department of Ageing Research, Manipal School of Life Sciences, Manipal Academy of Higher Education, Manipal, 576104 Karnataka India; 3 Department of Public Health Genomics, Manipal School of Life Sciences, Manipal Academy of Higher Education, Manipal, 576104 Karnataka India; 4 Department of Cell and Molecular Biology, Manipal School of Life Sciences, Manipal Academy of Higher Education, Manipal, 576104 Karnataka India; 5 Directorate of Research, Manipal Academy of Higher Education, Manipal, 576104 Karnataka India

**Keywords:** Neurodegenerative disorders, Co-exposure effect, Radiation exposure, Organophosphate pesticides, Lipid metabolism, Cholinesterase inhibitors, Neuroinflammatory diseases, Xenobiotics, Risk assessment, Biochemistry, Cell biology, Neuroscience, Systems biology, Zoology

## Abstract

Neurodegenerative disorders, such as Parkinson’s disease and Alzheimer’s disease, are major global health concerns and are linked to xenobiotic exposure. The rampant use of pesticides and increased number of radiological examinations can lead to neuronal alterations in the brain through oxidative stress and DNA damage. Understanding the impact of co-exposure to these agents can help identify interaction effects, enhance risk assessment, address vulnerable populations, and uncover long-term cumulative impacts that remain largely unknown. Therefore, in the current study, we aimed to explore the isolated and combined effects of low-dose radiation and malathion in the mouse brain. Mice were administered malathion (50 mg/kg) orally for 14 days, and a single whole-body low-dose radiation (0.5 Gy) on the 8th day. Five months post-exposure, behavioural, histological, enzymatic, and metabolomic analyses were carried out. Increased neuroinflammation and impaired neuronal maturation were observed in all treated groups, with neuronal death observed exclusively in the radiation group and persistent oxidative damage and acetylcholinesterase inhibition were identified in the malathion group. Additionally, the co-exposure group exhibited synergistic reductions in alpha-linoleic acid and linoleic acid metabolism, phosphatidylcholine biosynthesis, phospholipid biosynthesis, and sphingolipid metabolism within the hippocampus. Increased anxiety and reduced exploration were most pronounced in the co-exposure group, followed by the radiation group. This study provides insights into the effects of co-exposure to neurotoxicants such as low-dose radiation and malathion, revealing synergetic neuronal damage and dysregulated amino acid and lipid metabolism in the mouse hippocampus, and identifies metabolomic signatures enabling biomarker discovery and carries potential implications for the progression of neurodegeneration due to delayed systemic effects.

## Introduction

Neurodegenerative disorders are among the leading contributors to the global disease burden, with Parkinson’s disease (PD), Alzheimer’s disease (AD), and other types of dementia leading the race. The role of chemical, biological, or physical neurotoxic agents, such as pesticides, heavy metals, pollutants, radiation, etc., is well studied for their ability to cause neuronal alterations that lead to neurological disorders^1^. However, classical risk assessment strategies do not consider real-life exposure scenarios in which individuals are often exposed to multiple toxic agents^2^. The mechanism of action of a complex mixture can be synergistic or antagonistic^3^, and predicting the risks associated with such exposures can be challenging. Monitoring the effects of co-exposure can provide valuable biomarkers for predicting health effects and developing therapeutic strategies for treating these conditions.

The central nervous system (CNS) is susceptible to xenobiotic insults due to its high metabolic demand and reliance on postmitotic neurons to perform critical functions^4^. Importantly, the effects of exposure during early developmental stages, especially in children, may not become apparent until later in life^5^ with even minor disruptions potentially leading to neurotoxicity. Malathion (O, O-dimethyl-S-1,2-bisethoxy carbonyl ethyl phosphorodithionate) is a broad-spectrum organophosphate widely used in agriculture, domestic settings, and public health to increase crop yield and control disease-causing vectors (IARC^6^). Studies indicate that field sediments, wetlands, food, water, and breast milk have been found to have malathion residues well above the permissible limits^7,8^. In addition to being a cholinesterase inhibitor, malathion is known to induce oxidative stress, DNA damage, mitochondrial dysfunction, neuronal cell death, and alter many other neurological functions through noncholinergic mechanisms^9,10^. Furthermore, it is also known to cause alterations in crucial brain metabolites and hamper cellular signaling essential in neuronal maintenance and survival^11,12^.

Manned space missions pose risks to the CNS due to radiation exposure of about 0.1–0.5 Gy in the form of highly charged energy particles^13^. Globally, each year, roughly 3600 million radiological examinations, 7.5 million radiation procedures, and 37 million nuclear medicine procedures are performed (UNSCEAR^14^). Moreover, with increasing space missions, the radiation dose threshold of 600 mSv for astronauts set by the US National Academy of Sciences has raised concern, as several studies have proven detrimental effects at even lower doses^15^. These instances lead to exposure to several mGy of radiation, making it a health concern^13,16,17^. Prolonged exposure to low-dose radiation (LDR) over time induces neuroinflammation, diminishes neurogenesis and neuronal proliferation, resulting in various neurological sequelae^18,19^, including psychological consequences^20^. The resident glial cells, such as microglia and astroglia, are harbingers of neuroinflammation and play a key role in the pathophysiology of various neurodegenerative disorders^21^. The pathways influenced by high-dose radiation (HDR) differ from those affected by LDR, and therefore cannot be directly extrapolated to predict the potential impact of LDR^22^. There remains a knowledge gap regarding the impact of LDR on various pathological, genetic, and molecular aspects of the brain.

Metabolites regulate neuronal function through energy metabolism, altering neurotransmitter dynamics, oxidative stress modulation, and intercellular signaling^23^. Even slight biochemical shifts can influence synaptic activity, plasticity, and neuronal survival^24^. Studies have shown that disruptions in energy and amino acid metabolism in the brain can be linked to neurodegenerative disorder progression^25^. However, studies focusing on the effect of these exposures on hippocampal metabolomics do not exist, and therefore, focusing on elucidating the metabolomic signatures specific to this exposure can lead to the discovery of crucial biomarkers for neurodegenerative disease prediction. Therefore, it is essential to critically address the uncertainty surrounding the effects of LDR on the brain^26,27^.

LDR has been ambiguously shown to induce both harmful and adaptive neuronal responses^28–30^ while malathion has also been observed to induce neuronal damage^10^. Due to their overlapping mechanism of action, we wanted to assess if co-exposure to such agents induces synergistic or antagonistic reactions. Previously, we have observed alterations in neuronal functioning, survival, and death immediately after co-exposure^31^. However, long-term effects after exposure to these agents have not been studied.

Therefore, this study aimed to explore and compare the differences in neurotoxicity between isolated and combined exposure to two neurotoxicants, namely, LDR and malathion at an early age, and to determine whether combined exposure increases susceptibility to neurodegenerative-like effects over time. The present study thus serves as proof of concept to show that real-life co-exposures are more harmful than the standard single-agent study. Furthermore, a metabolomics-based approach was used to understand the changes in hippocampal biochemistry that drive these neurological alterations and the metabolomic pathways responsible for these changes.

## Results

### Behavioral assays

**Fig. 1:**
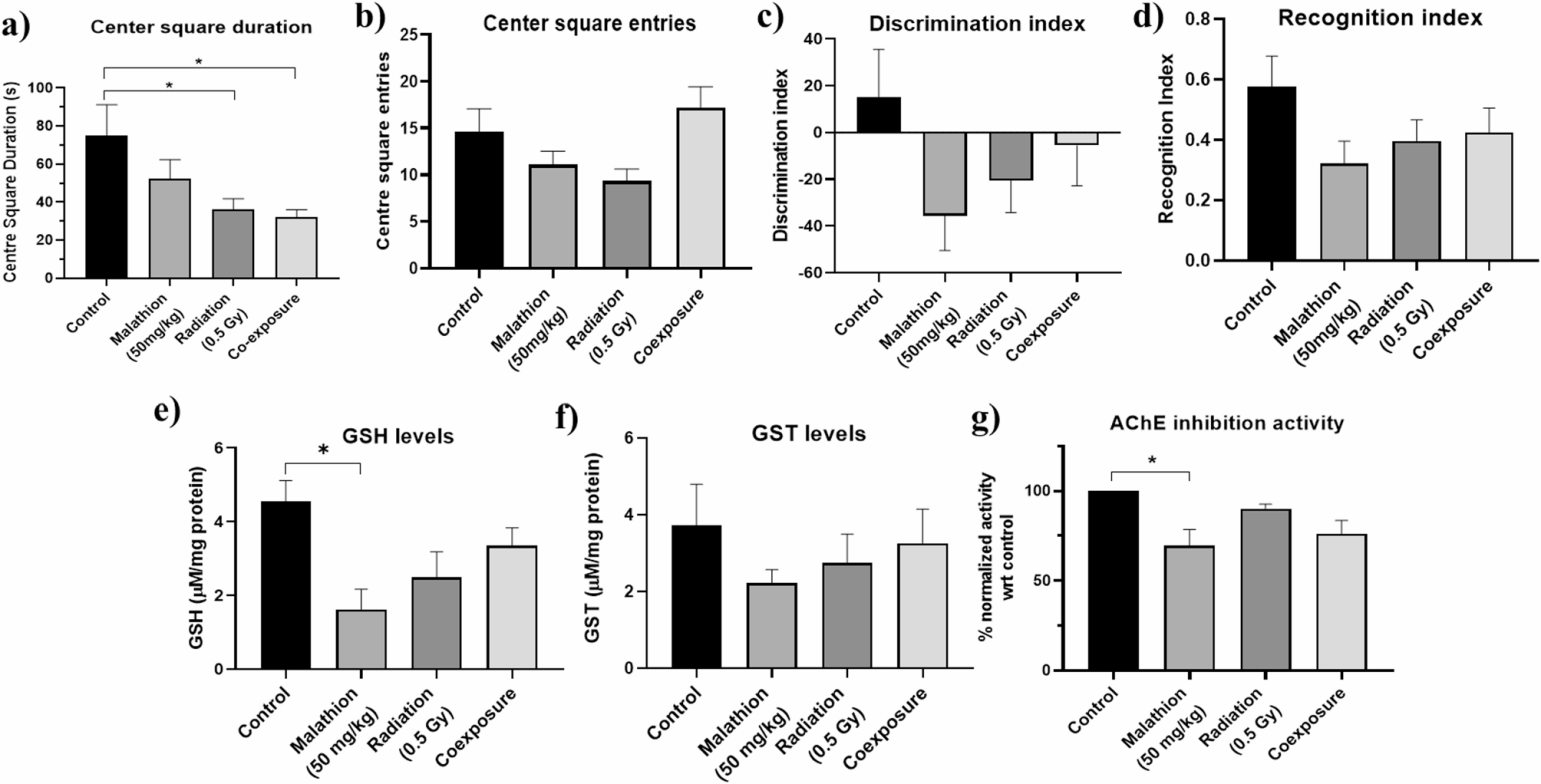
Effect of LDR and malathion single and co-exposure on behavior and enzymes. (**a**) Center square duration in the open field test; (**b**) center square entries in the open field test; (**c**) determination index in novel object recognition test; (**d**) recognition index in novel object recognition test (*n* = 9); Graphs depicting (**e**) GSH levels; (**f**) GST levels; (**g**) % acetylcholinesterase enzyme inhibition activity (*n* = 3); (*p < 0.05, data are represented as Mean ± SEM).

To evaluate exploratory behavior, we analyzed the duration and frequency of visits to the center square of the open field arena. The co-exposure and radiation groups displayed decrease in the time spent in the center (*p* < 0.05), suggesting reduced exploratory behavior. However, the number of times the animals entered the center square did not significantly vary between the groups (Fig. 1a,b). Despite observing a pattern of lower discrimination and recognition indices in the malathion, radiation, and co-exposure groups relative to the control, statistical analysis revealed that these reductions were not significant (Fig. 1c,d).

### Antioxidant capacity and acetylcholinesterase inhibition in the brain

Compared with the control group, only GSH (glutathione) levels were reduced in the malathion group (*p* < 0.01) (Fig. 1e). GST (glutathione S-transferase)levels were not altered across the treatment groups (Fig. 1f). Furthermore, AChE (acetylcholinesterase) inhibition significantly persisted (*p* < 0.05) only in the malathion group (Fig. 1g).

### Neuronal death and maturation

**Fig. 2:**
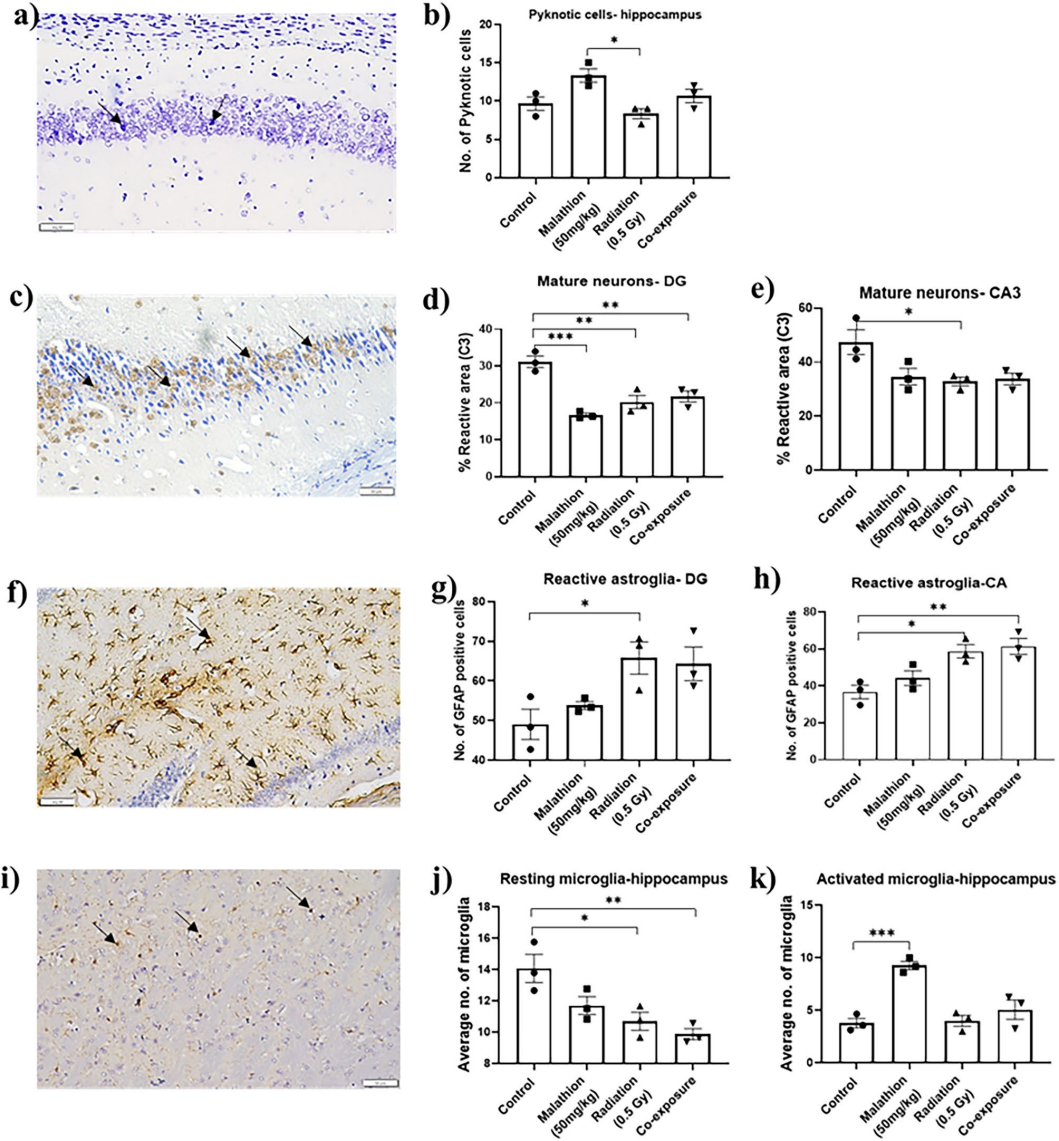
Effect of LDR and malathion single and co-exposure on neuronal survival, inflammation, and maturation. (**a**) Representative Nissl-stained mouse hippocampus; (**b**) Number of pyknotic neurons in different groups . (**c**) Representative image showing NeuN staining for mature neurons; (**d**) Percentage of NeuN-positive area in the DG region; (**e**) Percentage of NeuN-positive area in the CA3 region. (**f**) Representative image showing GFAP staining for astroglia; (**g**) Number of reactive astroglia in the DG region; (**h**) Graph representing the number of reactive astroglia in the CA region. (**i**) Representative image showing Iba-1 staining for microglia; (**j**) Number of resting microglia in the hippocampus; (**k**) Number of activated microglia in the hippocampus (*n* = 3, p< *0.05, **0.01, ***0.001, data represented as Mean ± SEM) (black arrow indicate positively stained cells, images in 40X).

Following exposure, we observed more pyknotic cells in the malathion group than in the radiation group (*p* < 0.05) (Fig. 2a,b). The rest of the groups presented levels comparable to those of the control. A decrease in NeuN expression (Fig. 2c) was observed in the malathion group (*p* < 0.001), followed by the radiation (*p* < 0.01) and co-exposure (*p* < 0.01) groups in the dentate gyrus (DG) (Fig. 2d). In the cornu ammonis (CA) 3 region, decreased expression was observed only in the radiation group (*p* < 0.05) (Fig. 2e).

### Persisting neuroinflammation

Increased astrogliosis was found in the DG region, only in the radiation group. In contrast, in the CA3 region, increased astrogliosis was found in both radiation (*p* < 0.05) and co-exposure groups (*p* < 0.01) (Fig. 2f,g,h). When assessing the hippocampal microglia (Fig. 2i), the number of resting microglia was found to be significantly lower in the co-exposure group (*p* < 0.01) and radiation group (*p* < 0.05) than in the control group (Fig. 2j). On the other hand, the number of activated microglia in the hippocampus was significantly higher in the malathion group (*p* < 0.0001) than in the control group (Fig. 2k).

### Metabolomic analysis

**Fig. 3:**
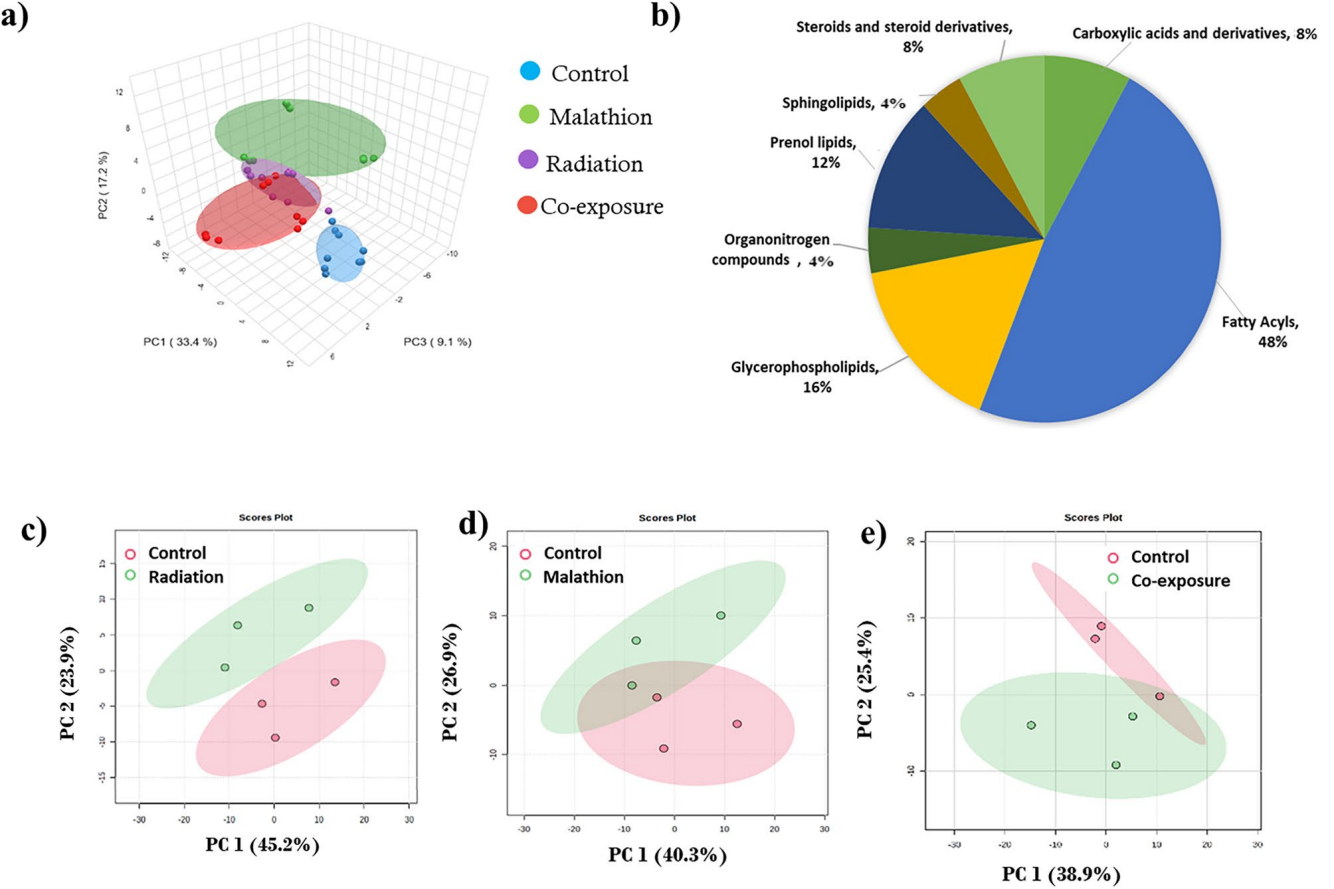
Hippocampal metabolome analysis. (**a**) PCA score plot of the variation between the samples of all four groups; (**b**) Pie chart showing the distribution of overall metabolites in different classes, with fatty acids representing the largest class, followed by glycerophospholipids and many others. PCA score plot of the variation between the samples of the - (**c**) Control and radiation group; (**d**) Control and malathion group; (**e**) Control and co-exposure group (*n* = 3).

We observed 1830 features, with 204 final features obtained for further analysis. Principal component analysis (PCA) of the whole metabolome profile indicated that the overall variation among all the groups in principal component (PC) 1, was 33.4% and that in PC2 was 17.2% (Fig. 3a). Furthermore, the majority of the metabolites altered post-exposure belonged to fatty acids, followed by glycerophospholipids, carboxylic acids and derivatives (Fig. 3b). In the individual PCA between the control and radiation treatments, PC1 and PC2 contributed 45.2% and 23.9% of variation, respectively (Fig. 3c), and between the control and malathion groups, PC1 and PC2 contributed to 40.3% and 26.9% of variation, respectively (Fig. 3d), indicating moderate separation from the control. The variation in the control and co-exposure stages was 38.9% in PC1 and 25.4% in PC2, suggesting that changes in the metabolome manifested over time in the co-exposure group (Fig. 3e).

### Metabolomic changes due to radiation exposure

**Fig. 4:**
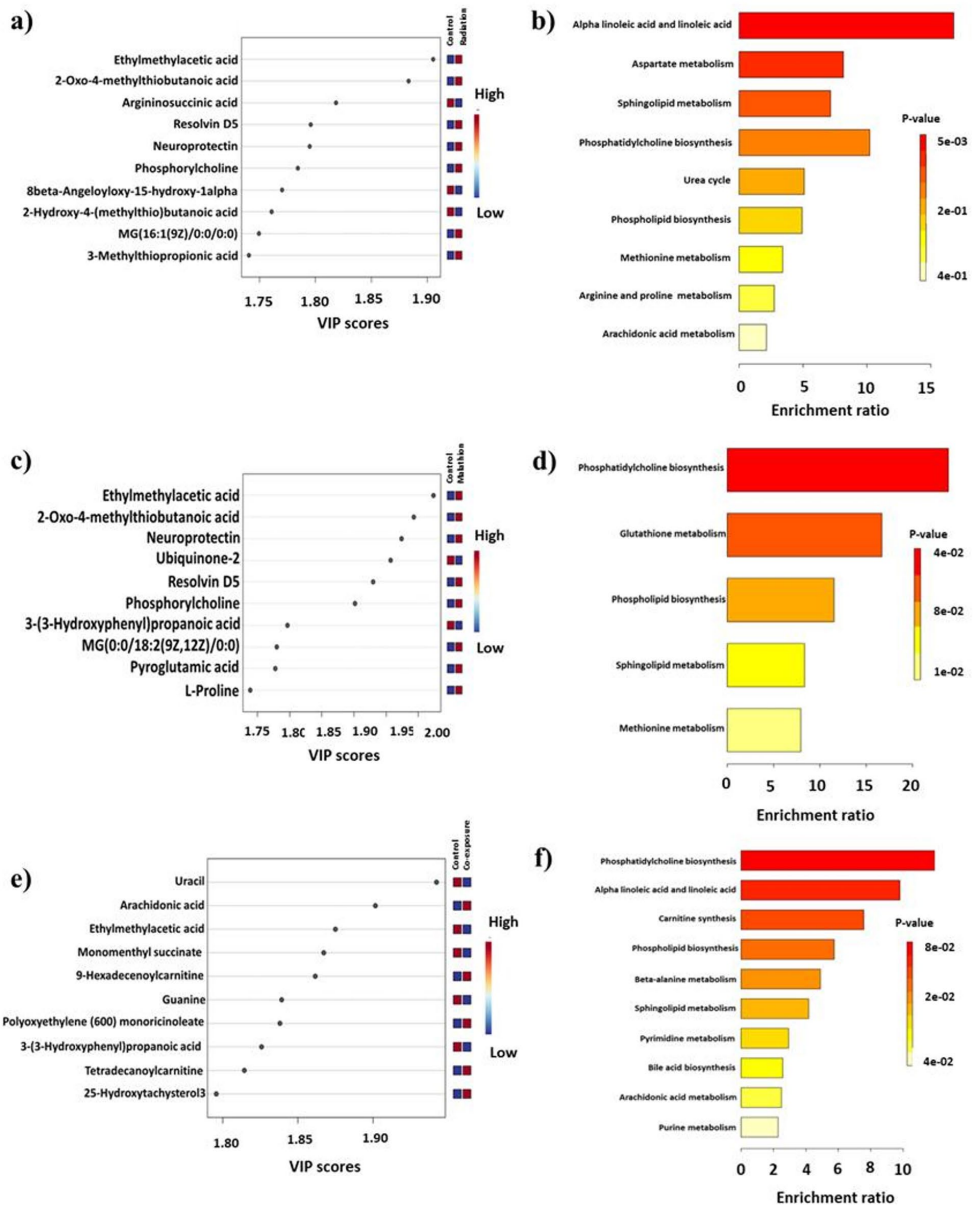
PLS-DA and pathway enrichment analysis, investigating metabolic alterations across different experimental conditions. (**a**) PLS-DA depicting the top 10 important metabolites in spectra post radiation exposure-several metabolites, including ethylmethylacetic acid, 2-oxo-4-methylthiobutanoic acid, argininosuccinic acid, resolvin D5, neuroprotectin, phosphorylcholine, and 3-methylthiopropanoic acid, exhibit high VIP scores; (**b**) Bar graph depicting enrichment values of the top significantly altered pathways after radiation exposure- alpha linolenic and linoleic acid metabolism, aspartate metabolism, sphingolipid metabolism, phosphatidylcholine biosynthesis, and the urea cycle show significant enrichment (**c**) PLS-DA depicting the top 10 important metabolites in spectra post malathion exposure-ethylmethylacetic acid, 2-oxo-4-methylthiobutanoic acid, neuroprotectin, ubiquinone-2, resolvin D5, phosphorylcholine, 3-(3-hydroxyphenyl)propanoic acid, MG(0:0/18:2(9Z,12Z)/0:0), pyroglutamic acid, and L-proline show high VIP scores; (**d**) Bar graph depicting enrichment values of the top significantly altered pathways after malathion exposure-phosphatidylcholine biosynthesis, glutathione metabolism, phospholipid biosynthesis, sphingolipid metabolism, and methionine metabolism were significantly enriched; (**e**) PLS-DA depicting the top 10 important metabolites in spectra post co-exposure-uracil, arachidonic acid, ethylmethylacetic acid, monomenthyl succinate, 9-hexadecenoylcarnitine, guanine, polyoxyethylene (600) monoricinoleate, 3-(3-hydroxyphenyl)propanoic acid, tetradecanoylcarnitine, and 25-hydroxytachysterol3 show high VIP scores; (**f**) Bar graph depicting enrichment values of the top significantly altered pathways after co-exposure-phosphatidylcholine biosynthesis, alpha linolenic and linoleic acid metabolism, carnitine synthesis, beta-alanine metabolism, sphingolipid metabolism, pyrimidine metabolism, bile acid biosynthesis, arachidonic acid metabolism, and purine metabolism were significantly enriched.

We observed 20 significantly upregulated and 13 significantly downregulated metabolites in the radiation group compared with those in the control group. Partial least squares-discriminant analysis (PLS-DA) revealed that the most discriminating feature was ethyl methyl acetic acid, with the remaining metabolites belonging primarily to the fatty acid class (Fig. 4a). The most altered pathway was involved in alpha-linoleic and linoleic acid metabolism, followed by aspartate metabolism, sphingolipid metabolism, phosphatidylcholine biosynthesis and the urea cycle. The top pathways altered due to radiation exposure are shown in Fig. 4b.

### Metabolomic changes due to malathion exposure

We obtained 13 significantly upregulated and 19 significantly downregulated metabolites in the malathion group compared to the control group, as assessed by t-test. Among these, the most discriminating feature was ethyl methyl acetic acid, with the remaining metabolites belonging to the metabolite class of carboxyl acids, derivatives, and fatty acids. Interestingly, ethyl methyl acetic acid was found to be the top discriminator of both the radiation and malathion groups. The top 10 distinct metabolites in the malathion group are depicted according to the variable importance in projection (VIP) scores in Fig. 4c. The most altered pathways were phosphatidylcholine biosynthesis, glutathione metabolism, phospholipid biosynthesis, sphingolipid metabolism, and methionine metabolism. Furthermore, we observed alterations in glutathione metabolism, corroborating our finding of persistent oxidative stress due to decreased antioxidant levels in the malathion group. The top pathways altered due to malathion exposure are shown in Fig. 4d.

### Metabolomic changes due to co-exposure 

We obtained 12 significantly downregulated and 21 significantly upregulated metabolites in the co-exposure group compared to the control group, as assessed by t-test. The most discriminating feature was uracil, which is involved in nucleotide metabolism, such as pyrimidine metabolism, beta-alanine metabolism, and pantothenate and coenzyme A biosynthesis. At the same time, the remaining metabolites belong to different metabolite classes, including carboxyl acids and derivatives, and fatty acids. The top10 distinct metabolites in the co-exposure group are depicted according to the VIP scores in Fig. 4e. Post-exposure, the five pathways most altered were phosphatidylcholine biosynthesis, alpha-linoleic and linoleic acid metabolism, carnitine synthesis, phospholipid biosynthesis, and beta-alanine metabolism. Like isolated exposure, co-exposure involved fewer alterations in amino acid metabolism and more alterations in various lipid and fatty acid metabolism pathways. The top pathways altered due to co-exposure are depicted according to their enrichment ratios in Fig. 4f.

### Comparison of metabolite levels between the groups

**Fig. 5:**
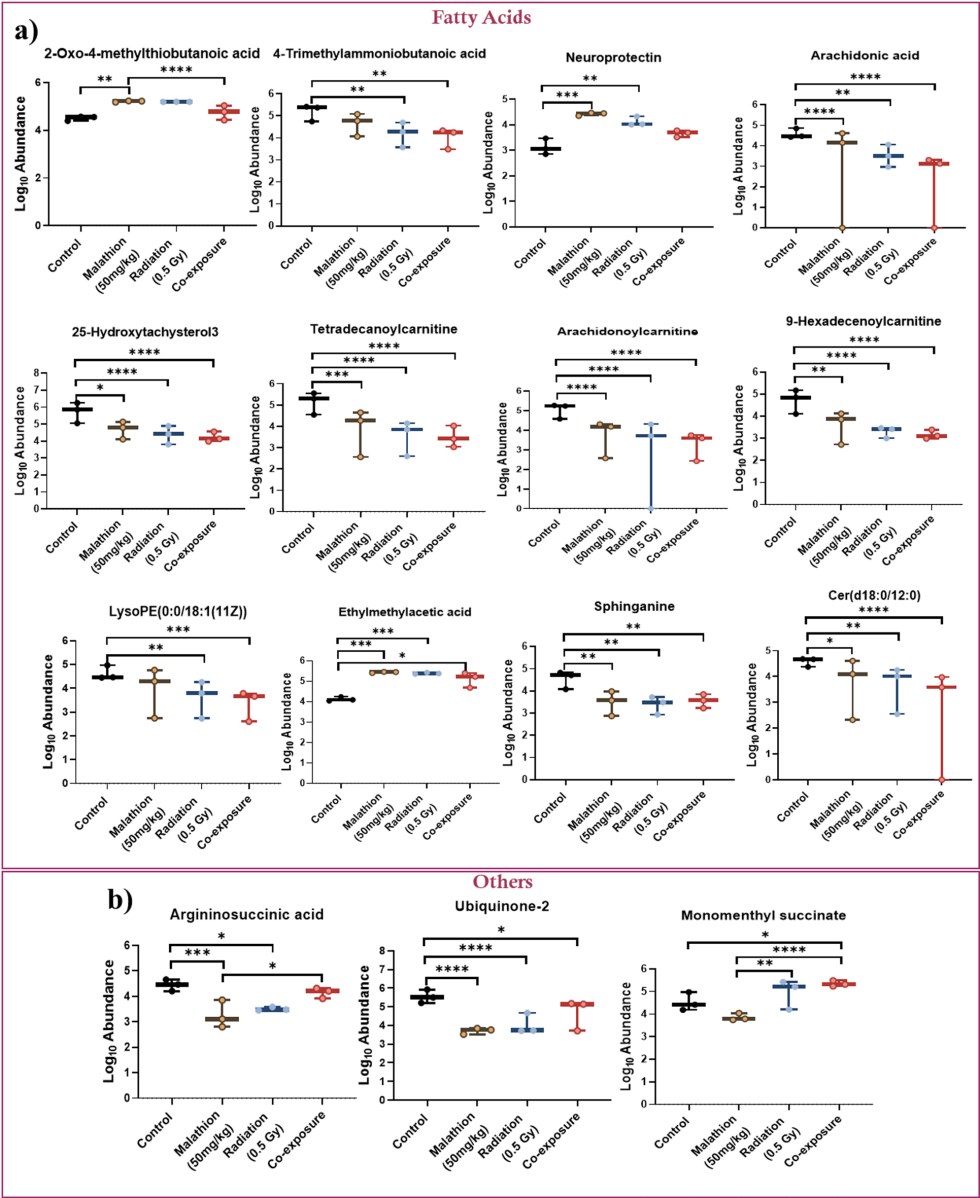
Altered metabolites of fatty acid metabolism and other pathways - (**a**) box and whisker plots reveal significant synergistic reduction in the abundance of several metabolites in the co-exposure group such as, -oxo-4-methylthiobutanoic acid, arachidonic acid, tetradecanoylcarnitine, arachidonoylcarnitine, 9-hexadecenoylcarnitine, and ceramide (d18:0/12:0); (**b**) panel b shows the impact of treatments on other metabolites, with argininosuccinic acid showing a synergistic reduction in the co-exposure (*n* = 3, error bar represents Mean ± SEM, *p < 0.05, **p < 0.01, ***p < 0.001, ****p < 0.0001).

**Fig. 6:**
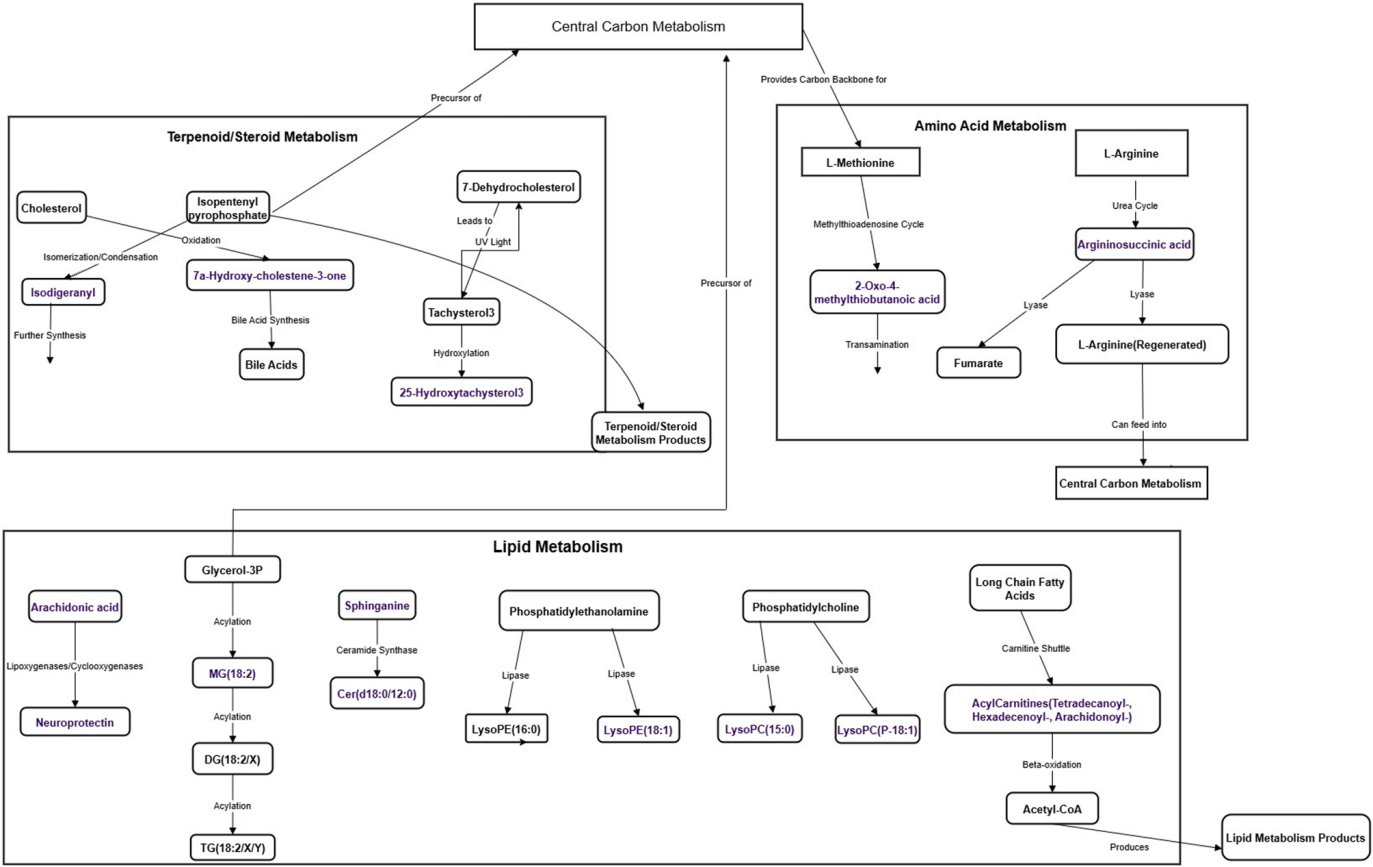
Overview of the metabolomic alterations and key pathways affected due to low dose exposure to radiation and malathion. The central carbon metabolism (CCM) serves as an energy source and provides precursors for various biosynthetic pathways like lipid metabolism, amino acid metabolism, and steroid metabolism, to name a few. Under the amino acid metabolism, the methylthioadenosine cycle can lead to the formation of 2-oxo-4-methylthiobutanoic acid, which can undergo transamination to form new amino acids. L-arginine’s involvement in the urea cycle leads to the formation of argininosuccinic acid, which ultimately regenerates back into L-arginine and is involved in the CCM again. Lipid metabolism broadly involves the metabolism of various lipids. Under the arachidonic metabolism, arachidonic acid gives rise to neuroprotectin due to the action of lipoxygenases. Under the glycerolipid metabolism, MG (0:0/18:2(9Z,12Z)/0:0) is produced through the acylation of glycerol-3-phosphate. Under the sphingolipid metabolism, ceramide (Cer(d18:0/12:0)) is synthesized from sphinganine through the action of ceramide synthase. Under the glycerolipid metabolism, phosphatidylethanolamine and phosphatidylcholine are broken down by lipases to give LysoPE(16:0), LysoPE(18:1), and LysoPC(15:0), LysoPC(P-18:1), respectively. Fatty acid beta-oxidation leads to the formation of different acylcarnitines from long-chain fatty acids. These then enter mitochondria and undergo beta-oxidation to produce acetyl-CoA, which can again enter CCM. Under the steroid metabolism, 7a-Hydroxy-cholestene-3-one is formed as an oxidation product of cholesterol and serves as a crucial intermediate molecule for the synthesis of bile acid. Further, the production of 25-Hydroxytachysterol3, which is a key metabolite of Vitamin D, is also observed. (The metabolites altered in our study are in purple font)

Notably, the levels of neuroprotectin, a long-chain fatty acid playing the role of a lipid messenger in the brain, and ethylmethylacetic acid, a saturated fatty acid, increased in the long-term effects post-radiation exposure. In contrast, the levels of 4-trimethylammoniobutanoic acid, arachidonic acid, sphinganine, argininosuccinic acid, monomenthyl succinate, ubiquinone-2, and many other fatty acid metabolites decreased (Fig. 5a,b). In animals exposed to malathion, metabolites such as neuroprotectin, ethylmethylacetic acid, and 2-oxo-4-methylthiobutanoic acid increased, whereas metabolites such as 9-hexadecenoylcarnitine, 25-hydroxytachysterol3, arachidonoylcarnitine, argininosuccinic acid, sphinganine, ubiquinone-2, tetradecanoylcarnitine, and metabolites of the fatty acid class decreased (Fig. 5a,b). Interestingly, the levels of most of the metabolites altered in the isolated exposure scenario were further reduced in the co-exposure scenario, indicating that synergistic effects were particularly pronounced after a long-term period (Fig. 5a,b). The overall mechanistic overview of the altered metabolic pathways with their respective metabolites is depicted in Fig. 6. The list of altered metabolites in different groups and their respective abundance values, log2 fold change, and p-values are listed in Table 1. Further, the overall alterations and the pathological manifestations are depicted in Fig. 7.

**Table 1:** List of metabolites significantly altered in different exposure settings compared to those in the control group, along with their respective abundance and log2 fold change values.

Metabolite	Metabolite class	Radiation (log2 fold change)	Radiation (adjusted *p*-value^#^)	Malathion (log2 fold change)	Malathion (adjusted *p*-value^#^)	Co-exposure (log2 fold change)	Co-exposure (adjusted *p*-value^#^)
Ethylmethylacetic acid	Fatty acids	4.1749	0.0003	4.3481	0.0001	3.5077	0.0086
2-Oxo-4-methylthiobutanoic acid	Fatty acids	2.2571	ns	2.3257	0.0044	1.0038	ns
4-Trimethylammoniobutanoic acid	Fatty acids	-2.9239	0.0106	-2.2911	0.0057	-3.3089	0.0187
Monomenthyl succinate	Prenol lipids	1.7137	ns	-2.5661	ns	2.3496	0.0386
Isodigeranyl	Prenol lipids	-2.9348	0.0288	-2.6542	0.0331	-2.8045	0.0274
Sphinganine	Organonitrogen compounds	-3.8310	0.001	-3.2052	0.0033	-3.3786	0.0076
Argininosuccinic acid	Carboxylic acids and derivatives	-3.2365	0.0123	-3.3273	0.0007	-1.0124	ns
Arachidonic acid	Fatty acids	-3.0194	0.0029	-1.1996	< 0.0001	-5.2272	< 0.0001
Polyoxyethylene (600) monoricinoleate	Fatty acids	-1.6520	ns	-0.3622	ns	-4.1804	0.0002
MG (0:0/18:2(9Z,12Z)/0:0)	Fatty acids	2.7484	< 0.0001	3.3947	< 0.0001	2.1781	< 0.0001
Tetradecanoylcarnitine	Fatty acids	-4.7981	< 0.0001	-3.2279	0.0001	-5.3735	< 0.0001
3-Hydroxy-cis-5-tetradecenoylcarnitine	Fatty acids	-1.6439	< 0.0001	-1.7221	< 0.0001	-4.5885	< 0.0001
9-Hexadecenoylcarnitine	Fatty acids	-5.1480	< 0.0001	-3.4624	0.0013	-5.6131	< 0.0001
Neuroprotectin	Fatty acids	3.1341	0.0071	4.0674	0.0002	1.5646	ns
25-Hydroxytachysterol3	Steroids and steroid derivatives	-4.5393	< 0.0001	-3.6039	0.0045	-5.4520	< 0.0001
7a-Hydroxy-cholestene-3-one	Steroids and steroid derivatives	-4.8954	< 0.0001	-3.8310	0.001	-6.2886	< 0.0001
Arachidonoylcarnitine	Fatty acids	-3.9531	< 0.0001	-3.4550	< 0.0001	-5.3422	< 0.0001
LysoPE (0:0/16:0)	Glycerophospholipids	-1.8271	0.0404	-0.7469	ns	-2.9614	0.0032
LysoPE (0:0/18:1(11Z))	Glycerophospholipids	-2.5913	0.0046	-0.9432	ns	-3.7607	0.0002
LysoPC (15:0/0:0)	Glycerophospholipids	-1.9500	< 0.0001	-0.3247	< 0.0001	-3.69945	< 0.0001
Cer (d18:0/12:0)	Sphingolipids	-2.0610	0.0087	-1.1537	0.0188	-3.1579	< 0.0001
LysoPC (P-18:1(9Z)/0:0)	Glycerophospholipids	-0.4833	ns	-3.0377	0.0258	0.7593	ns

#- p-values were calculated using two-way ANOVA.

**Fig. 7:**
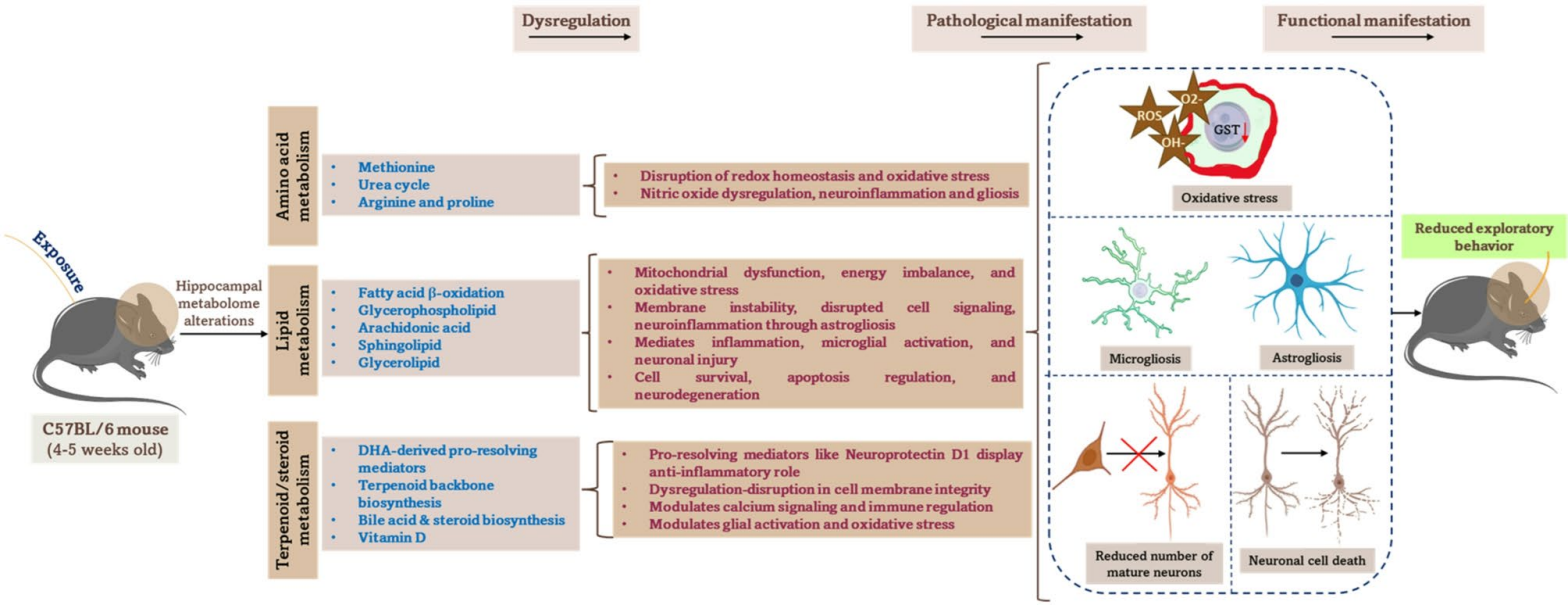
Schematic illustration of the hippocampal metabolomic and cellular alterations in mice exposed to low-dose radiation and malathion (isolated and combined) exposure. Isolated and co-exposure of low dose malathion and radiation lead to dysregulation in amino acid, lipid, and steroid metabolism pathways. These metabolomic disruptions lead to redox imbalance, oxidative stress, neuroinflammation, gliosis, and altered cellular signaling. These responses can collectively result in long-term functional impairments such as reduced exploratory behavior.

## Discussion

The neurotoxic effects of radiation and pesticides are well documented, but the molecular mechanisms underlying low-dose exposure, especially over the long-term, remain poorly understood. This study investigated the mechanisms contributing to neurotoxic and potential neurodegenerative effects caused by combined exposure to radiation and malathion.

Our results revealed sustained oxidative damage after malathion exposure, as evidenced by reduced GSH levels, consistent with the findings of other studies in rodents showing variations in antioxidant levels^32,33^. Free radical production and oxidative stress have been linked to neurodegenerative diseases^34^. Additionally, we observed neuroinflammation through astroglial activation in the radiation and co-exposure groups and microgliosis in the malathion-treated group, corroborating previous findings that malathion affects pro-inflammatory markers such as IL-6 and TNF-α^35^. In addition to Iba-1, elevated expression of neuroinflammatory markers such as Cd68 and Cd11c upon radiation exposure is also reported^36^. Reports also indicate increased microgliosis and astrogliosis in mice after radiation exposure (0.125 Gy and 0.5 Gy)^37^. In individual exposures, while a significant increase in activated microglia was observed only in the malathion group, their levels were reduced in the radiation group. A similar reduction was also seen in the co-exposure group. This antagonistic interaction observed between radiation and the malathion group may be attributed to the modulatory effect of low-dose radiation on microglial activation induced by the malathion. This effect is likely due to the low radiation dose and the extended time interval between exposure and assessment, which may have induced a hormetic response in microglia, leading to their desensitization and the development of immune tolerance^38^.

Persistent oxidative stress and neuroinflammation led to cell death in the malathion-treated group, supporting earlier studies^39^ and a reduction in the number of mature neurons was consistent with these findings. Previously, we observed neuronal cell death as an immediate consequence of exposure to malathion in the mouse hippocampus^31^. Moreover, malathion has been shown to induce apoptotic cell death through lysosomal membrane permeabilization in the mouse neuroblastoma cell line N2a^40^, indicating that in addition to oxidative stress and neuroinflammation, this could also be one of the mechanisms behind the observed cell death in our study.

Lipids, constituting about 50% of the brain’s dry weight, are crucial to the brain, particularly for neuronal energetics, and their disruption is known to affect neuronal transmission, dendritic morphology, spine density, etc^41^. Particularly, the synaptic junctions possess an abundance of lipid and fatty acid transporters that bind to polyunsaturated fatty acids that drive essential functions such as synaptic transmission and synaptic plasticity^42^. Therefore, even a subtle change in the composition and content of these lipid molecules can disrupt neurotransmission, alter cellular signaling, and predispose an individual to various neurodegenerative disorders mediated through synaptic dysfunction^43^. Our study has revealed that sphingolipid metabolism is one among the several lipid metabolism pathways altered due to both individual and co-exposure to low-dose radiation and malathion. Studies have shown that synaptic junctions are enriched with lipid molecules such as cholesterol and sphingolipids that control the membrane viscosity and serve as substrates for the biosynthesis of oligodendrocytes and myelin sheath^44^. Therefore, alterations in the sphingolipid metabolism may negatively affect the mobility of crucial molecules across the lipid membrane, thereby affecting neuronal transmission.

In the radiation group, metabolomic analysis revealed changes in lipid and fatty acid metabolism in the hippocampus, with ethyl methyl acetic acid being notably altered. Further, amino acid metabolism pathways, such as aspartate metabolism, arginine and proline metabolism, and methionine metabolism, were also altered post-exposure. Amino acids are crucial for neurotransmitter synthesis and also act as precursors for several other metabolisms, with their dysregulation being linked to several neuronal pathologies^45^. Aspartate metabolism, which was seen altered in our study, has previously been known to involve protein synthesis, metabolism of several other amino acids like phenylalanine, tyrosine, methionine, threonine, lysine and isoleucine, and even enter other metabolic pathways like ornithine cycle, urea cycle and nucleotide synthesis etc^45^. On the other hand, altered arginine metabolism has been linked to tauopathy and other neuronal disorder-associated pathology^46^. Proline, essential for protein synthesis, is also known to regulate oxidative homeostasis and apoptosis, with a crucial role in cognition^47^. Methionine, a key precursor for the synthesis of cysteine, homocysteine, carnitine, and other essential metabolites, and plays a role in lipid metabolism as well as activates the synthesis of antioxidant enzymes such as methionine sulfoxide reductase and glutathione^48^. Arachidonic acid accounts for about 12% of the fatty acids in the brain and is crucial for cognition and synaptic transmission, and the decreased turnover of arachidonic acid metabolites has been reported in psychiatric diseases^49^.

Some of the altered metabolites in the radiation group included 3-oxadecanoic acid, alpha-linolenic acid, valeric acid, phospholipids, acylcarnitines, etc., all of which are components of various lipid metabolism pathways. Aberrant lipid metabolism is proposed to underlie denervation of neuromuscular junctions, mitochondrial dysfunction, excitotoxicity, impaired neuronal transport, cytoskeletal defects, inflammation, and reduced neurotransmitter release^42^. As an intermediate of long-chain fatty acid synthesis, 3-oxodecanoic acid plays a crucial role in brain lipid metabolism^50^. Another metabolite, valeric acid, is a crucial anti-inflammatory molecule that can protect cells from undergoing oxidative stress and autophagy in the brain^51^. Acyl carnitines, like 4,8-dimethylnonanoyl carnitine, which was found altered in our study post-radiation exposure, are known to impart neuroprotection^52^. Another crucial metabolite, alpha-linoleic acid, which was seen to be altered, is an omega-3 fatty acid that plays a crucial role in neurogenesis, synaptic transmission, calcium signaling, and cognition^53^. Previously, radiation exposure in rats has been shown to increase lipid peroxidation, decrease membrane fluidity, and alter the lipid-to-protein ratio^54^ which could explain the lipid metabolism disruptions observed here. Given the role of lipids in cell signaling, our findings suggest that altered signaling pathways may be a result of lipid metabolism changes.

In the malathion group, pathway enrichment revealed shifts in fatty acid and lipid metabolism, including the phosphatidylcholine biosynthesis, phospholipid, and sphingolipid metabolism pathways. Research has shown that pesticides disrupt lipid metabolism, neuroinflammation, mitochondrial function, receptor activity, and signal transduction^12^. Previous studies done on mouse liver post-malathion exposure have reported alterations in fat, protein and energy metabolism^55^. Interestingly, we observed altered glutathione metabolism, which plays an important role in maintaining redox homeostasis and imparting antioxidant defense, and its depletion or dysregulation is one of the hallmarks of several neurological disorders^56^. The depletion of GSH observed by us in the present study in the malathion group may be metabolically regulated by the altered glutathione metabolism observed.

Key metabolites such as ethylmethylacetic acid, MG (0:0/18:2(9Z,12Z)/0:0), neuroprotectin, and resolvin D5 belonging to the fatty acid class were seen to be altered post-malathion exposure. Neuroprotective compounds such as neuroprotectin and resolvin D5 are autocoid proteins that mitigate inflammation and promote neuronal survival^57^. Generally, in disorders such as AD, neuroprotectin 1 levels are seen to be reduced; however, their increased level in the brain promotes the survival of neurons by increasing the expression of antiapoptotic genes^58^. Further, resolvins offer neuroprotection by the suppression of pro-inflammatory cytokines like IL-6 and TNF-α and inhibit microglial activation^57^ which might explain the observed decrease in microglial expression that we have observed in our study. Like radiation exposure, malathion exposure too saw an alternation of a metabolite pimelylcarnitine, belonging to the group of acylcarnitine and it is seen in significantly lower levels in patients with AD^59^ and higher levels are associated with lesser risk of the disease^60^.

Compared to isolated exposure, co-exposure resulted in synergistic alterations in a few of the metabolites associated with lipid and fatty acid metabolism. Lipids and fatty acids, which supply 20% of the brain’s energy, play a role in synaptic signaling^47^. Over time, alterations in these metabolites may contribute to age-related cognitive decline via blood-brain barrier disruptions, signaling pathway alterations, mitochondrial dysfunction, oxidative stress, and gut-brain axis disruptions^61^. Under steroid metabolism, 7a-Hydroxy-cholestene-3-one is formed as an oxidation product of cholesterol and serves as a crucial intermediate molecule for the synthesis of bile acid, which is one of the pathways altered in co-exposure. Bile acids have been known to play a role in neuronal transmission and regulate brain physiology^62^. The PLS-DA analysis highlighted uracil variability, a component of nucleotide metabolism, as well as pantothenate and CoA biosynthesis, which can disrupt DNA/RNA precursor balance and lead to replication errors and DNA damage^63^. Moreover, uridine, a derivative of uracil, can act as a neurotransmitter and participate in several neuronal processes, including memory and plasticity^64^. Apart from this, guanine, a component of purine metabolism that was seen to be altered in co-exposure, is an essential modulator of neuronal signaling and acts as a neurotrophic agent in the brain^65^. The rest of the metabolites that were seen to be altered belonged to fatty acid metabolism, comprising carnitines, and their role in maintaining neuronal health has already been emphasized in the previous sections. Apart from this, a significant reduction in the other carnitines like arachidonoyl carnitine, tetradecanoyl carnitine, and 3-hexadecenoylcarnitine was also observed in all three groups, with co-exposure showing the highest reduction. Previously, these carnitines have been known to prevent neurological disorders such as AD^66^.

From the metabolomic data obtained in the study, it can be said that there are potential shifts in the metabolic flux through different lipid metabolism pathways, like sphingolipid metabolism, phospholipid metabolism, glycerophospholipid metabolism, etc. This, in turn, is connected closely to energy metabolism and hence can lead to altered flux in glycolysis and the TCA cycle, thus affecting the energy availability for neurons to carry out their functions. Further, decreased levels of lipid metabolites can also affect lipid membranes and transport. Moreover, these lipids act as signaling molecules, and hence, the variation in their levels can alter crucial pathways like inflammatory signaling, which could be one of the reasons for the observed neuroinflammation in the exposure groups. However, despite noticing the synergistic reduction in a few metabolites, we acknowledge that when considering the overall number of metabolites detected, the number of statistically significant metabolites remains modest.

Behavioral assays revealed reduced exploratory behavior in the radiation and co-exposure groups, whereas the malathion group showed a similar but non-significant trend. Previous studies have shown that exposure to low-dose radiation leads to reduced performance in various behavioral tests , such as impaired memory in novel object recognition, worse performance, and increased latency to visit the island in the Morris water maze test^67,68^. This highlights that behavioral impairments may manifest over a long period in low-dose exposure scenarios. However, contradictory findings of low-dose radiation resulting in improved performance through antidepressant-like effects and improved performance in open field tests and forced swimming tests have also been reported^69^. In the present study, although we noticed a reduced trend in the discrimination and recognition indices, this trend was not significant in the novel object recognition test. This implies that there might be a slight suppression of behavior, and may represent early neurofunctional disruptions that precede more pronounced impairments, which may, after cumulative wear and tear, manifest as overt pathology past a certain threshold^70,71^. Further, several reasons could potentially inhibit the observed metabolomic synergism from becoming translatable functional changes. For example, there may be several compensatory cellular mechanisms and pathways in place in the brain to buffer untoward changes. Further, metabolomic changes may be early indicators of behavioral changes that can be expected over the long term, especially in low-dose studies.

Future studies involving fractionated radiation doses, long-term exposures, both sexes, and multiple time points could provide more information on the exposure of a more complex nature. A chronic, dose-response study would help us better dissect and correlate the neuronal and metabolomic shifts associated with different doses and timepoints. A more thorough investigation into many other behavioral tests focusing on diverse cognitive aspects could improve our understanding of functional manifestations of these cellular and molecular changes. While we have incorporated technical replicates and statistical approaches to overcome the limitation of a smaller sample size, to fully compensate for biological variability, it would be beneficial if the study were carried out in larger cohorts in the future.

## Conclusion

In the present study, we have shown that exposure to malathion and radiation, individually or in combination, affects overlapping biological processes and pathways crucial for hippocampal neuronal function. Further, metabolomic analysis suggests that co-exposure can induce synergistic biochemical alterations in some of the brain fatty acid and lipid metabolites, with crucial neuronal implications and potentially contributing to neurodegenerative disease mechanisms. Our findings underscore the importance of limiting exposure to neurotoxicants such as malathion in domestic and procedural settings involving radiation. Furthermore, co-exposure to neurotoxicants at a younger age could potentially accelerate the neurodegenerative symptoms observed in the exposed individuals, highlighting the varied interaction of co-exposure to multiple neurotoxic agents as opposed to individual exposure. Studying the co-exposure effects at lower doses over extended periods could reveal neuronal damage persistence under single and repeated exposure conditions, and chronic scenarios may reveal delayed-onset effects due to gradual brain damage accumulation.

To our knowledge, this is one of the preliminary studies reporting on the metabolomic profile of the hippocampus in such low-exposure scenarios, and it can influence future work in the realm of environmental toxicology and systems biology. Further, it lays the groundwork for more targeted research into the most abundantly altered pathways reported, thereby establishing early biomarkers of neurotoxicity. In particular, molecules like arachidonoylcarnitine, neuroprotectin, sphinganine, and cer(d18:0/12:0), etc., are involved in the lipid-mediated regulation of neuroinflammation and apoptotic pathways that could have impacts on oxidative damage, microglial activation, and neuronal survival. Utilizing the findings of the present study to perform a more targeted metabolomic analysis will further help delineate accurate biomarkers of neurotoxicity. Further, quantitative validation of these metabolites in blood and CSF across exposed populations can provide us with an established metabolite panel that could aid in early diagnosis and disease monitoring. Moreover, conducting functional studies on enzymes involved in these metabolomic pathways could reveal any dysregulation in feedback loops or specific bottlenecks. Additionally, our study highlights the need for co-exposure models, which remain a relatively underexplored area despite the real-world relevance attached to them. We show that combined exposure can lead to unpredictable interactions and impact in a much distinct manner compared to that of individual exposures, calling for the need for further such studies.

## Methodology

### Animals and treatment conditions

The study utilized male C57BL-6 J mice obtained from the Central Animal Research Facility at Kasturba Medical College, Manipal Academy of Higher Education, Manipal, after receiving approval from the Institutional Animal Ethics Committee (IAEC/KMC/108/2019). All the methods and experimentation were performed according to the relevant regulations and guidelines, including the ARRIVE guidelines. The mice were housed in sterile polypropylene cages under controlled environmental conditions, with an optimal temperature of 20 ± 2 °C and a 10-hour light/14-hour dark cycle. They had ad libitum access to sterile food and water. Thirty-six male animals, aged four to five weeks and weighing approximately an average of 19.4 ± 1.6 g, were randomly divided into four groups, each containing nine animals: control, malathion (50 mg/kg orally in saline), ionizing radiation (IR, 0.5 Gy), and co-exposure (50 mg/kg malathion orally + 0.5 Gy IR).

Taking into account the dose exposed during manned space missions (0.1–0.5 Gy)^13^ and during radiotherapy fractions (0.15 –0.2 Gy)^16^and further considering the repeated exposure scenario, we chose 0.5 Gy for our study. Integrated Risk Information System (IRIS) Chemical Assessment Summary from the US Environmental Protection Agency reported that the malathion dose of 50 mg/kg was established as the lowest dose level at which neurological consequences, such as acetylcholinesterase inhibition, were noted in mice. Further, several studies have reported high residues of malathion detected in various food items like rice, vegetables, etc., with concentrations as high as 18.26 mg/kg and above the permissible limits^72–74^. According to WHO, the acceptable daily dose of malathion is 0-0.3 mg/kg b.wt., which, when converted to mice after applying relevant conversion factors^75^ is around 3.6 mg/kg b. wt., making our choice of dose approximately 14 times the acceptable intake. Hence, we chose a dose of 50 mg/kg/ body weight as the dosage for malathion treatment.

Behavioral assays were conducted for nine animals per group, with three mice per group used for the rest of the experiments. Previous transcriptomic^76^ and proteomic^77^ studies have similarly employed three animals per group to depict pilot findings post xenobiotic exposure and diet changes, respectively. Specifically, other neuronal studies have also used three animals for histological and molecular investigations^78–80^. Furthermore, we obtained multiple technical replicates per mouse to enhance the robustness of the data. This approach balances the need for statistically meaningful results with ethical considerations, minimizing animal use in accordance with the 3Rs principle.

**Fig. 8:**
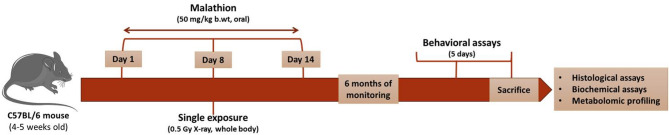
Experimental design depicting the study timeline in C57BL/6 male mice. Mice were subjected to 14 days of daily oral malathion administration (50 mg/kg body weight), with a single whole-body X-ray exposure (0.5 Gy) on the 8th day. After the exposure period, animals were monitored until they reached six months of age, and followed by behavioral testing animals were sacrificed for histological analysis, biochemical assays, and hippocampal metabolomic profiling.

Malathion (96% purity, MP Biomedicals, USA) was administered orally via gavage for 14 consecutive days. On the 8th day, a single whole-body exposure to 0.5 Gy of X-ray radiation was carried out, following the procedure outlined previously^81^. Body weight was recorded weekly until the end of the study, followed by behavioral tests. The animals were sacrificed five months post-exposure through cervical dislocation according to the American Veterinary Medical Association guidelines, and fresh brains were collected for histological and molecular assays. For histological analysis, the samples were stored in 10% neutral buffered formalin immediately following the dissection. For the metabolomic analysis, the fresh hippocampus was isolated on ice and immediately snap frozen using liquid nitrogen and stored at -80 ˚C until further processing as described under sample preparation for mass spectroscopy. The overall treatment timeline is represented in Fig. 8.

### Behavioral assays

#### Open field test for anxiety-related behavior

The animal was positioned at the center of an open square field, facing away from the experimenter, and allowed to explore freely for 5 min while being video recorded. After 5 min, the animal was removed, and the field was sanitized with 70% alcohol and allowed to dry. The coded video was subsequently analyzed, and the time spent in the center and the number of entries into the center square were recorded^82^.

#### Novel object recognition test for memory evaluation

On the first day, the animals were habituated to the testing arena for ten minutes. During the familiarization phase, the animals were given at least 20 s to explore two identical objects. After 24 h, one of the objects was replaced for the retention phase. The test was video recorded, and the data were analyzed by an observer blinded to the experimental conditions. Key metrics, including the duration and frequency of exploration of the novel and familiar objects, were recorded. The discrimination index (DI) and recognition index (RI) were then calculated. DI was determined via the following formula: (time spent exploring the novel object – time spent exploring the familiar object)/(time spent exploring novel + familiar) * 100, whereas RI was calculated as the time spent exploring the novel object/total time spent exploring both objects^83^.

### Antioxidant and acetylcholinesterase (AChE) enzyme inhibition assay

Assays for GSH, GST antioxidant enzymes, and AChE inhibition were carried out as described previously^84,85^. For the biochemical assays, 10% tissue homogenates were prepared using a lysis buffer (0.01 M Tris HCL (pH 7.4), 1 M NaCl, 0.01 M EGTA, 1% Triton X-100 made up to 100 mL), and protein concentration was measured using Bradford assay. The standards for GST and GSH were prepared by dissolving reduced glutathione in phosphate buffer (PB) (81 mL of 0.2 M K_2_HPO_4_, 19 mL of KH_2_PO_4_ adjusted to pH 7.4 in 100 mL).

For the GSH assay, the samples were incubated with 5% w/v metaphosphoric acid for 2 h at 4 °C, and further, the supernatant was obtained by centrifugation at 12,000 rpm for 5 min. The samples (250 µL) were mixed with 0.06 mM of DTNB and 0.2 M of PB to make up to a volume of 1 mL and incubated for half an hour at 37 °C. The color developed because of the reaction between the GSH and 5,5-dithiobis-2-nitrobenzoic acid was measured at 412 nm. The concentration of the GSH was determined from the standard plot.

For the GST assay, an assay cocktail of 980 µL of PB, 10 µL of 100 mM CDNB prepared in ethanol, and 10 µL of Glutathione was prepared. For sample estimation, 900 µL of this enzyme cocktail was mixed with 100 µL of sample, and the reaction between GST and 1-chloro, 2, 4-dinitrobenzene was measured at 340 nm for 5 min. The concentration of the GST was calculated using the molar extinction coefficient of CDNB (0.0096 µM^−1^/cm) using the formula-.

GST activity = [(Adjusted Δ340/min)/ 0. 0096 µM-1/cm] x (1.0 ml /0.1 ml) x sample dilution

For the AChE assay, 180 µL of DTNB was mixed with 10 µL of the sample and 10 µL of the substrate. The reaction between thiocholine of the substrate acetylthiocholine iodide and 5,5-dithiobis-2-nitrobenzoic acid was measured kinetically for 5 min at 412 nm. The absorbance/minute using the slope was calculated, and the activity was calculated using the formula-.

Rate = (Absorbance minute)/ 1.36 × 104, where 1.36 × 104 is the extinction coefficient of the yellow anion (5-thio-2-nitrobenzoic acid)

The activity in the treatment group was normalized to that of the control group. The values of all three assays were normalized with protein concentration and expressed in terms of µM/mg protein for GSH and GST, and percentage normalized activity with respect to the control for AChE.

### Histology

The entire brain was fixed in 10% neutral buffered formalin (NBF) and embedded in paraffin. Sectioning was performed via a rotary microtome (Leica Biosystems, Germany) to obtain 5 μm sections collected on slides coated with poly-L-lysine.

### Nissl staining for neuronal survival

Nissl staining was carried out as previously described^86^. Briefly, sections were rehydrated in different alcohol grades and distilled water. The sections were then stained in cresyl violet, followed by dehydration, clearing, and mounting. Images were captured via an Olympus CKX53 microscope (Olympus, Japan), and image analysis was performed via Fiji software’s Cell Counter plugin to score pyknotic cells. Neurons were identified by their relatively larger size, the presence of abundant dark granular cytoplasm with clumped Nissl granules, and a distinct nucleolus. In contrast, pyknotic cells were identified by their similar size and uniform dark staining. Three animals per group, and three hippocampal sections per animal, were analyzed.

### Immunohistochemistry

Immunohistochemistry was performed for the markers NeuN, Iba-1, and GFAP as previously described^31^. Briefly, 5 μm sections were collected on charged slides and subjected to treatment with xylene and rehydration in different grades of alcohol and distilled water. Antigen retrieval was carried out with sodium citrate buffer, followed by blocking with 10% normal serum containing 1% BSA in PBS. The antibodies NeuN, Iba-1, or GFAP in 1% BSA in PBS were incubated overnight with the sample, followed by washing and peroxidase activity blocking. The secondary antibody was treated for one hour. The signal was developed using DAB chromogen (Dako, USA) with hematoxylin as a counterstain. The sections were then dehydrated, cleared, mounted, and observed using DPX. For each group, three animals were used, with a minimum of three images from three sections captured for analysis. The images were captured via an Olympus CKX53 microscope (Olympus, Japan), and the analysis of Iba1- and GFAP-expressing cells was conducted via Fiji software along with the Cell Counter plugin. NeuN expression was quantified as the percentage of the positively stained area^87^.

### Metabolomic analysis

Sample preparation for LC-MS, mass spectrometric run conditions, and subsequent data analysis were carried out for the hippocampal tissues (*n* = 3) in triplicate with a ppm error limit of 15 as previously optimized^81^. Briefly, following exposure, mice were sacrificed, and 20 mg of hippocampal tissue was taken, to which, around 1 mL of methanol: water at a ratio of 4:1, was added to facilitate protein precipitation. The samples were subjected to three cycles of vortexing (30 s), submerged in liquid nitrogen (2 min), thawed for 3–4 min, and sonicated for 5 min at 30s intervals with a 5 s pause. The samples were then centrifuged at 12,000 rpm at 4 °C for 10 min, and the supernatant was subjected to lyophilization. The residue was reconstituted in 100 µL of cold acetonitrile and water (1:1, v/v) containing 1% formic acid and again subjected to centrifugation at 12,000 rpm for 10 min, and the supernatant was stored at -80 °C until analysis.

Mass spectrometry was carried out using ESI-QTOF (Agilent 6250 TOF-MS, Agilent Technologies, USA) combined with a high-performance liquid chromatography system (Agilent 1200 series, USA). Prior to injection, the samples were reconstituted in 100 µL of solvent containing water and acetonitrile with 0.1% formic acid. A 5 µL sample was injected into an analytical column (ZORBAX Eclipse XDB C18, 4.4 × 250 mm, 5 microns) and the run was carried out in positive mode for 45 min each. A mobile phase consisting of acetonitrile and water with 0.1% formic acid was used, and a gas flow of 8 L/min, a gas temperature of 250 °C, nebulizer pressure of 40 psig, and ESI capillary voltage of 3500 V, were maintained during the run.

All the raw data analysis, as well as statistical multivariate analysis like principal component analysis and partial least squares-discriminant analysis, were performed through the automated tool Metaboanalyst 5.0. The raw data was obtained in the .d (data dependent acquisition) format and converted to mzML format using MS Convert (ProteoWizard) software^88^ and uploaded to Metaboanalyst. The data was filtered based on the standard deviation, and normalization by median was carried out, followed by logarithmic transformation. Statistical analysis between the two groups employed a student’s t-test, while the comparison of the levels of each metabolite between all four groups was carried out using two-way ANOVA. Principal component analysis, variable importance of projection, and pathway enrichment analysis were carried out within Metaboanalyst.

### Statistical analysis

The results were statistically analyzed using GraphPad Prism Version 8 via tests such as t-test, one-way ANOVA, and two-way ANOVA. For comparison between the two groups, particularly in metabolomic analysis, a t-test was used. A comparison of the control group, simultaneously with the other three treatment groups, was carried out using one-way ANOVA (behavioral tests, biochemical estimations, and histological observations). Two-way ANOVA was used to analyze the effect of treatment and the different metabolites against the metabolite abundance levels. Data were expressed as means ± SEM to estimate variability between means of multiple trials in each sample. A p-value of < 0.05 was considered to indicate statistical significance.

## Data Availability

The datasets generated during and/or analyzed during the current study are available from the corresponding author on reasonable request.
